# The Influence of Thermal Annealing on the Chemical Composition, Nanomechanical, and Nanotribological Properties of Tantalum Thin Films

**DOI:** 10.3390/mi16040427

**Published:** 2025-04-02

**Authors:** Debottam Datta, Ali Eskandari, Junaid Syed, Himanshu Rai, Nitya Nand Gosvami, Ting Y. Tsui

**Affiliations:** 1 Department of Materials Science & Engineering, Indian Institute of Technology Delhi Hauz Khas, New Delhi 110016, India; debottamdatta555@gmail.com (D.D.); syed.junaid@mse.iitd.ac.in (J.S.); er.anshurai@gmail.com (H.R.); 2 Department of Chemical Engineering, University of Waterloo, 200 University Avenue West, Waterloo, ON N2L 3G1, Canada; ali.eskandari@uwaterloo.ca; 3 Waterloo Institute for Nanotechnology, University of Waterloo, Waterloo, ON N2L 3G1, Canada

**Keywords:** tantalum, nanotribology, wear, friction, hardness, elastic modulus

## Abstract

Tantalum metal and tantalum oxide thin films are commonly used in semiconductor devices, protective coatings, and biomedical implants. However, there is limited information on their nanotribological behavior and small-scale mechanical properties. This study characterized the chemical, mechanical, and tribological properties of as-deposited and 400 °C annealed β-Ta thin films using nanoindentation and atomic force microscope (AFM)-based nanoscale friction and wear tests. X-ray photoelectron spectroscopy (XPS) results revealed that a thermally grown Ta oxide layer forms on the surface of Ta film after being annealed at 400 °C. The nanoindentation data indicated an increase in both the hardness and elastic modulus in the heat-treated sample compared to the as-deposited Ta film (13.1 ± 1.3 GPa vs. 12.0 ± 1.4 GPa for hardness) and (213.1 ± 12.7 GPa vs. 175.2 ± 12.3 GPa for elastic modulus). Our nanotribological results show that the friction increased and wear resistance decreased on the surface of the annealed sample compared to the as-deposited Ta film. This discrepancy may be caused by the oxidation of Ta on the film surface, which induces residual compressive stresses in the film and degrades its wear resistance. Our results highlight the influence of thermal annealing and oxidation on nanotribological behavior and small-scale mechanical properties of Ta thin films.

## 1. Introduction

Tantalum (Ta) and its alloys are widely used in engineering applications, such as semiconductor integrated circuits [1,2,3], biomedical implants [4,5,6,7,8], and protective hard coatings [9,10]. These materials exhibit excellent mechanical strength, wear resistance, diffusion barrier, and biocompatibility. Ta thin films can be prepared in the alpha-Ta (α-Ta) phase with a body-center-cubic (BCC) crystalline structure and in the beta phase (β-Ta) with a tetragonal crystal structure. The mechanical properties of Ta thin films often depend on fabrication techniques, deposition conditions, and post-deposition treatments [11,12,13,14,15,16,17,18,19,20,21]. Generally, α-Ta is softer than β-Ta. The hardness of α-Ta films reported in the literature is in the range of ~6.55 and ~12 GPa [11,12,14,15,16,18], while the β-Ta counterpart ranges from ~11.3 to ~23 GPa [11,15,16,17,18,21,22]. The deposition methods and conditions also influence the elastic modulus of Ta films. They can be between ~123.4 and ~260 GPa for α-Ta [12,14,15,16,18], and ~188 to ~250 GPa for β-Ta [15,16,17,18,21].

As Ta films are mechanically robust, thin protective layers are often used to enhance the tribological properties of numerous material surfaces, such as stainless steel [23], aluminum alloys [24], and rubbers [25]. Pathote et al. [26] reported that the wear rate of the biomedical grade 316 L stainless steel substrate could be reduced by more than ten times when protected with a Ta thin film deposited using DC magnetron sputtering. The effectiveness of Ta in providing tribological protection varies and depends on its phase composition. Su et al. [16] demonstrated that the hard metastable tetragonal β-Ta coating offers better wear resistance and adhesion on Ti–6Al–4V substrates than the soft α-phase Ta.

In addition to Ta metal, Ta oxide thin films are widely used in engineering devices due to their high mechanical strength [24] and biocompatibility [27]. Ta oxide thin films can be thermally grown or prepared using various physical vapor deposition techniques. The hardness and elastic modulus of Ta oxide reported in the literature range from ~5.3 GPa to ~14.6 GPa [28,29,30,31,32] and from ~108.1 GPa to ~420 GPa [28,29,31,33], respectively. One common application of Ta oxide film is as a wear protection layer. For example, Ding et al. [27] coated the biomedical Ti–6Al–4V substrate surface with a multilayer structure consisting of reactive sputtered Ta oxide, Ta oxide-titanium oxide, and Ta. This multilayer coating reduced the friction force and enhanced wear resistance compared to the pure Ta oxide and bare Ti–6Al–4V surface. Generally, the wear performance of Ta oxide film improves with its density [27,30,34,35], which can be enhanced with post-deposition annealing. Rahmati et al. [35] demonstrated that the densification of Ta oxide can be achieved through thermal annealing at temperatures above 300 °C for 60 min. Their scratch test results show that the Ta oxide film annealed at 500 °C exhibits a critical load of failure from the scratch test that is more than twice that of the as-deposited film.

While the prior studies [16,23,26,27] focused on the wear behaviors of Ta and Ta oxide on a macroscopic scale, where film cracking and delamination are the dominant failure mechanisms, the mechanical deformations related to the nanoscale tribological wear of Ta films remain limited. In nanotribology, plastic deformations often involve removing materials on an atomic scale rather than through macroscopic failure modes. The nanotribological performance and wear behavior on as-deposited and annealed Ta and Ta-oxide, such as material removal rate and friction during nanometer scale contacts, are highly critical for the success of chemical mechanical polishing (CMP) and planarization in advanced integrated circuit fabrication and biomedical implant devices.

The primary objective of this work is to investigate the chemical composition and small-scale mechanical and tribological properties of Ta thin films subjected to thermal treatments. X-ray photoelectron spectroscopy (XPS) results indicate that a thin native Ta oxide forms on the as-deposited Ta film surface. This oxide layer thickness increases with post-deposition anneal at 400 °C. Nanoindentation results revealed that the hardness and elastic modulus values of the annealed Ta film are larger than those of the as-deposited Ta film. Contrary to the nanoindentation results, the nanotribological properties measured using an atomic force microscope (AFM) show that the annealed Ta film surface was more prone to wear when compared with the as-deposited Ta film, and the friction force measured on the annealed film was higher than on the as-deposited specimen. This is believed to be the first study to reveal this annealing-driven nanoscale wear degradation effect on the tribological properties of Ta films. Herein, we investigated how the post-deposition annealing affects the nanomechanical and nanotribological properties of Ta thin film.

## 2. Materials and Methods

### 2.1. Ta Metal Deposition and Annealing

Ta thin films with a thickness of 150 nm were deposited onto silicon substrates in the G2N cleanroom laboratory at the University of Waterloo, Waterloo, Canada. The deposition was conducted using an AJA ORION series sputtering tool (AJA International, Hingham, MA, USA). It operated at a DC power of 100 W with a base chamber pressure maintained at 5 mTorr. Argon gas was flown into the deposition chamber at a rate of 12 sccm, resulting in a Ta deposition rate of ~5 nm/min. We have chosen the sputtering deposition technique due to its widespread application in preparing Ta thin films in the semiconductor manufacturing industry and biomedical implant devices. Following sputter deposition, the Ta film was annealed at 400 °C for 30 min in a tube furnace purged with high-purity nitrogen (99.998% purity). The furnace temperature rose from ambient to 400 °C in about 10 min and the cooling process was completed in ~20 min.

### 2.2. X-Ray Photoelectron Spectroscopy

The chemical composition of the Ta film specimens was characterized using X-ray photoelectron spectroscopy (XPS) depth profiling techniques (Axis Supra, Kratos Analytical Ltd., Kyoto, Japan). The instrument is equipped with an aluminum Kα X-ray beam at a take-off angle of 90°, and the measurement chamber was maintained at a high vacuum of 1 × 10^−7^ Torr. Sputtering steps during profiling experiments were conducted with an argon ion beam operating at 5 keV with an incident beam angle of 40°.

### 2.3. X-Ray Diffraction

The structural properties of the as-deposited and annealed Ta films were determined using the grazing incident angle X-ray diffraction (GIXRD) technique at the incident angle of 0.5°. The experiment was performed using a commercial X-ray diffractometer (Rikagu Ultima IV, Tokyo, Japan, Cu Kα, λ: 1.54 Å) in an ambient atmosphere.

### 2.4. Nanomechanical Characterization

The hardness (H) and elastic modulus (E) of fused silica, a bare silicon substrate, as-deposited Ta thin film, and Ta film annealed at 400 °C were characterized using a nanoindenter that operates in a continuous stiffness measurement mode [36]. Fused silica served as both reference and calibration material. During the indentation experiments, a sharp Berkovich diamond indenter tip oscillated at a frequency of 75 Hz with a harmonic displacement of 1 nm. A strain rate of 0.05 s^−1^ was maintained throughout the loading segment with a targeted maximum indenter tip penetration depth of 300 nm. The load, displacement, and contact stiffness data were analyzed using the Oliver and Pharr methods [36] to compute the nanoindentation H and E values.

### 2.5. Nanotribology of Ta Films

The nanotribological behavior of as-deposited and annealed Ta films was characterized using an atomic force microscope (FlexAFM, Nanosurf, Switzerland) operating in the lateral force microscopy (LFM) mode. The AFM experiments were performed with diamond-coated silicon probes (All-In-One-DD, BudgetSensors, Bulgaria). The cantilever beam had a normal spring constant of 100 N/m, as determined by using the Sadar and Green method [37,38]. For friction and wear measurements, the diamond tip rastered and slid on the specimen surface with increasing normal load within a square-shaped area measuring 2 × 2 μm^2^. After the test, a zoomed-out image was acquired below 100 nN normal load to measure wear within the experimental region where friction vs. load tests were performed. The sampled square area was divided into 256 equally spaced lines, and the AFM tip slid back and forth on each line at a scan rate of 20 μm/s. The lateral deflection of the cantilever beam during the sliding contacts was recorded during both the forward (trace) and backward (retrace) motion. The lateral deflection signal was then converted to friction force by multiplying the voltage signal with the lateral force sensitivity and by dividing the difference between the forward and backward signals by two, which helps nullify the vertical offsets in the friction loops caused by laser misalignment. Customized MATLAB (version R2020a) scripts were utilized to analyze the friction vs. load and wear volume data and calculate the volume of materials removed by the diamond tip during the wear experiment. All the AFM images were processed using the commercial scanning probe microscopy software Gwyddion (version 2.61).

## 3. Results and Discussions

### 3.1. Chemical Composition of Ta Films

**Figure 1:**
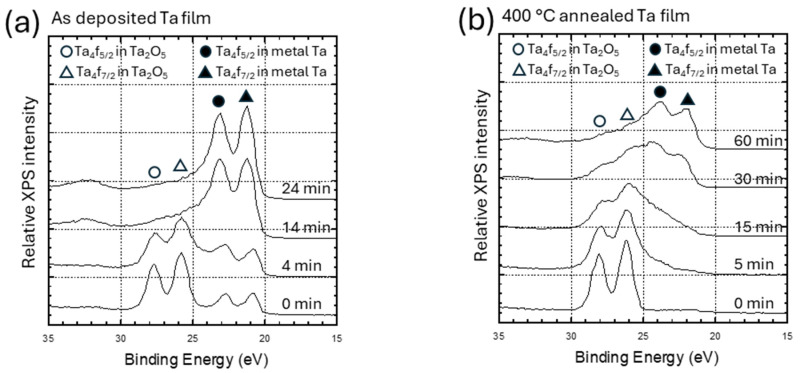
Relative XPS spectrum of (**a**) as-deposited Ta film and (**b**) 400 °C annealed Ta film as a function of sputtering time. The results show that the intensity of Ta_4_f_5/2_ and Ta_4_f_7/2_ peaks in Ta_2_O_5_ decreases with the sputtering time. In contrast, the Ta_4_f_5/2_ and Ta_4_f_7/2_ peaks in metallic Ta increase when films are sputtered. This indicates that the sputtering process removes the surface oxide and exposes the Ta metal film.

XPS depth profiling techniques were used to characterize the chemical composition of the Ta films. The Ta peaks, specifically Ta_4_f_5/2_ and Ta_4_f_7/2_ [3,27,39], associated with tantalum pentoxide (Ta_2_O_5_) and Ta metal, were analyzed and are displayed in Figure 1. Figure 1a illustrates the Ta_4_f_5/2_ and Ta_4_f_7/2_ peaks for Ta_2_O_5_ and Ta metal from the surface of the as-deposited sample. These results indicate that a thin layer of native Ta oxide was present on the surface of the as-deposited film prior to Argon (Ar) sputtering. Notably, the intensity of Ta_2_O_5_-related Ta_4_f_5/2_ and Ta_4_f_7/2_ peaks diminishes with prolonged sputtering time while the Ta metal peak increases. After 24 min of sputtering, the Ta_2_O_5_ peak features disappear from the spectrum, leaving only those associated with Ta metal. This observation indicates that the kinetic bombardment of Ar ions and the possible reduction of the Ta oxide during Ar sputtering [40] have removed the Ta oxide layer, exposing the Ta metal layer beneath.

The XPS spectrum for the Ta film annealed at 400 °C is displayed in Figure 1b. The results indicate that the surface before sputtering displayed Ta_4_f_5/2_ and Ta_4_f_7/2_ peaks associated with Ta_2_O_5_, while no signals from metallic Ta were detected. This suggested that the surface was initially composed entirely of Ta oxide and contained no metallic Ta prior to Ar sputtering. It took ~60 min of sputtering to completely remove the Ta oxide layer from the annealed Ta specimen. Interestingly, this time is nearly twice as long as ~24 min required to remove the native oxide from the as-deposited film. These findings suggest that the thermally grown Ta oxide thickness is approximately twice that of the native Ta oxide found on the as-deposited film. Our results align with those reported by Cheng et al. [39], who observed a 46% increase in Ta_2_O_5_ layer thickness and a 12% increase in density after a 10 min anneal at 400 °C.

### 3.2. Crystalline Structure of Ta Films

**Figure 2:**
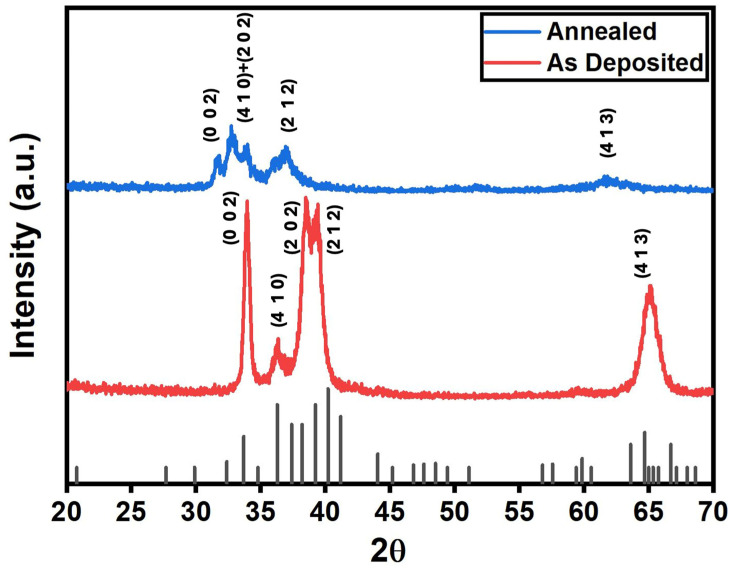
Grazing-incidence angle X-ray diffraction plots for as-deposited (red spectrum) and 400 °C annealed (blue spectrum) Ta film. It shows the as-deposited film is predominately composed of β-Ta.

The crystalline structures of the as-deposited and annealed films were characterized using GIXRD technique at the incident angle of 0.5°. Figure 2 displays the diffraction spectra of the as-deposited (red spectrum) and 400 °C annealed (blue spectrum) Ta films. The as-deposited film exhibits strong β-Ta signals corresponding to (002), (410), (202), (212), and (413) peaks of the known β-Ta data of ICDD 00-025-1280. The XRD results indicate that the crystalline structure of the as-deposited Ta film is comparable to those prepared by other researchers using similar fabrication techniques [18,39,41,42]. However, the peaks are slightly shifted towards the higher θ value by ~0.5° due to in-plane stress variation, attributed to the sputtering pressure at which the Ta film is deposited. This finding is consistent with the results of Ellis et al. [20] where the peaks of β-Ta film are shifted due to the variation in Ar pressure. The diffraction spectrum of the films annealed at 400 °C is also presented in Figure 2 where β-phase Ta peaks are shifted towards a lower θ value, attributed to the increase in d-spacing [43,44] caused by the volumetric expansion of Ta film due to surface oxidation [45].These findings are consistent with the results of Cheng et al. [39] for annealed Ta films. Further, Chandrasekharan et al. [46] illustrated that the peaks of Ta film can be shifted towards the lower θ value due to surface oxidation. The broadening of the peaks and reduced intensity observed in our annealed sample may be due to signal interference from the thick, thermally grown Ta oxide layer on the specimen surface, as indicated by the XPS results. The crystallite size of the as-deposited and annealed Ta film was calculated using the Williamson–Hall (W-H) method [47]. The results revealed that the crystallite size was 22 ± 0.3 nm and 4.6 ± 0.25 nm for the as-deposited and annealed Ta films, respectively.

### 3.3. Nanomechanical Properties of the As-Deposited and Annealed Ta Films

**Figure 3:**
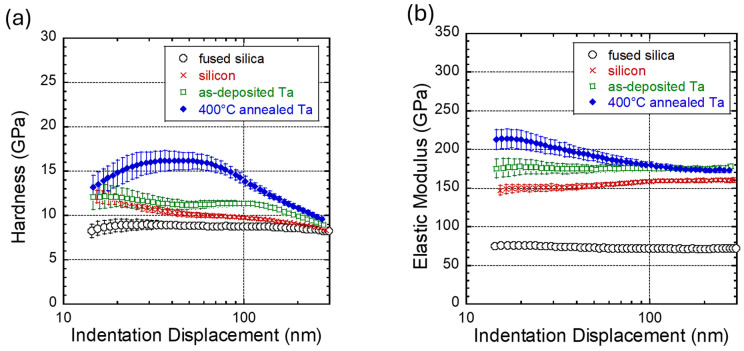
Plots of nanoindentation (**a**) hardness and (**b**) elastic modulus of fused silica, silicon substrate, as-deposited Ta thin film, and 400 °C annealed Ta film as a function of indentation depth. Results show that the hardness and elastic modulus of Ta film increase significantly after thermal annealing.

The hardness and elastic modulus of bulk fused silica, bare silicon substrate, and Ta thin films were measured using nanoindentation techniques, with the fused silica serving as the reference material. Figure 3 presents the results of the indentation measurement, plotting the hardness and elastic modulus as a function of indentation depth. The data spread in the figures corresponds to one standard deviation. As shown in Figure 3, the hardness and elastic modulus of the Ta thin films approach those of the silicon substrate at large indentation depths. Measurements were taken at smaller indentation depths of 15 nm or 10% of the film thickness to obtain more representative properties of the Ta films. For the as-deposited Ta film, the results indicate that the hardness and modulus at these small depths are similar to those of the silicon substrate. In contrast, the hardness and modulus of the annealed Ta film increase considerably at small depths. Interestingly, Figure 3a shows that the hardness of the annealed Ta film reaches a maximum value of 16.2 ± 1.0 GPa at indentation depths of ~41 nm before decreasing at small depths. This indentation depth-dependence behavior of film hardness suggests a softer surface layer on the annealed Ta film, which likely contains Ta_2_O_5_ based on our XPS analyses in Section 3.1. This thin oxide surface layer may play a more critical role in nanoscale wear than the underlying Ta film, which appears harder and has a hardness of at least 16.2 ± 1.0 GPa.

The hardness and elastic modulus values of the four specimens are summarized in Table 1. The fused silica and silicon results presented in this table are the average values calculated from measurements at indentation depths between 15 nm and 300 nm. Results show that the hardness and elastic modulus of fused silica are 8.7 ± 0.2 GPa and 72.8 ± 1.6 GPa, respectively. They are comparable with literature values of 8.77 ± 0.02 GPa [48] and 73.3 GPa [49]. The silicon substrate has hardness and elastic modulus values of 10.0 ± 1.0 GPa and 155.6 ± 4.2 GPa, respectively. The hardness and modulus of Ta films reported in Table 1 were obtained from measurements at indentation depths near 15 nm (or ~10% of the film thickness). Table 1 shows that the as-deposited and annealed Ta films have an elastic modulus of 175.2 ± 12.3 GPa and 213.1 ± 12.7 GPa, respectively. These values are comparable with those of β-Ta thin films reported by other researchers of ~188 GPa to ~250 GPa [15,16,17,18,21]. The 175.2 ± 12.3 GPa elastic modulus of our as-deposited Ta film is statistically indistinguishable from those reported by Abadias et al. [15] of 188 ± 3 GPa and comparable to the measurements of sputter deposit Ta films reported by Guisbiers et al. [50] of 175 ± 3.4 GPa.

Our indentation results also reveal that the annealing process increased the small depth hardness of the as-deposited Ta film from 12.0 ± 1.4 to 13.1 ± 1.3 GPa. These results aligned well with those reported in the literature for β-Ta films in the range of ~11.3 and ~23 GPa [11,15,16,17,18,21,22], and for Ta oxide films ranging from ~5.3 GPa to ~14.6 GPa [28,29,30,31,32]. The increase in the small depth hardness and modulus of the Ta film after annealing can be attributed to the rise of the surface layer film density [13,27,30,35,51].

### 3.4. Nanotribological Properties of As-Deposited and Annealed Ta Films

**Figure 4:**
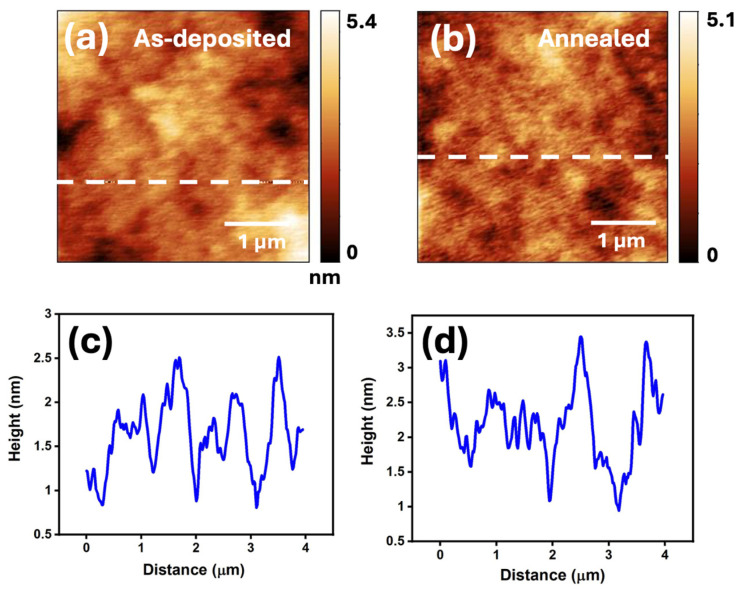
Surface morphology of the (**a**) as-deposited and (**b**) annealed Ta film before performing the friction test; (**c**) the line profile of the white dash line marked on figure (**a**); (**d**) the line profile of the white dash line marked on figure (**b**).

While the macro- and micro-scale wear properties of as-deposited and annealed Ta films have been studied previously, limited information exists regarding their nanotribological properties. Herein, we used an AFM-based technique to investigate the surface topography and nanotribological properties of both as-deposited and annealed Ta thin films. Prior to conducting the nanotribological experiments, the surface topographic features on the as-deposited and annealed Ta films were characterized by AFM. The corresponding images are illustrated in Figure 4a,b, respectively. The cross-sectional line profiles of the surfaces along the dash lines indicated in these figures are shown in Figure 4c,d. The results show that the surfaces are considerably smooth with roughness on the nanometer scale. Furthermore, Table 2 indicates that there is no significant change in the surface root-mean-square (RMS) roughness and the mean roughness before and after the 400 °C annealing. The surface roughness was measured in 2 × 2 μm^2^ area for both the surfaces.

**Figure 5:**
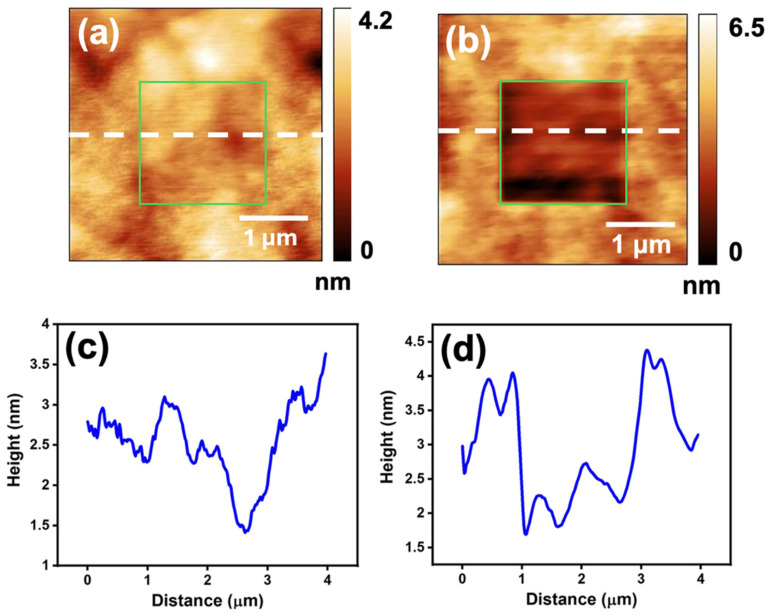
Worn out regions after performing friction test on the surface of (**a**) as-deposited and (**b**) annealed tantalum film up to the normal load of 5 μN; (**c**) the line profile of the white dotted line marked on figure (**a**); (**d**) the line profile of the white dotted line marked on figure (**b**). The green boxes indicate the sampled areas of the wear test.

**Figure 6:**
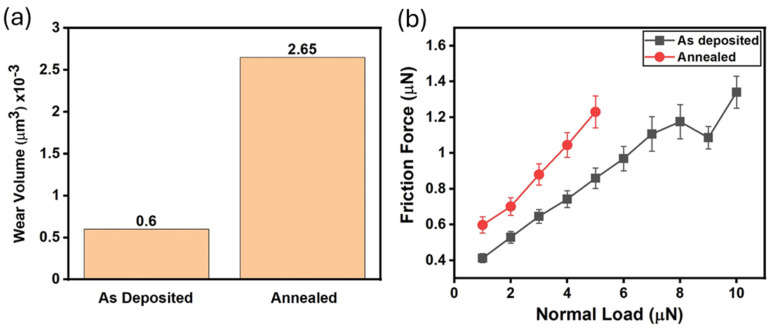
(**a**) A plot of calculated wear volume for the as-deposited and annealed tantalum film; (**b**) friction force with increasing normal loads in both the as-deposited and annealed tantalum films.

Wear experiments were conducted within a square-shaped region measuring 2 × 2 μm^2^ using a sharp diamond tip. The surface topographic features of the worn areas, under a normal load of 5 μN, on the as-deposited and annealed Ta surfaces were imaged by AFM techniques, as shown in Figure 5a,b. The green boxes in these figures highlight an area of ~2 × 2 μm^2^ and indicate the worn regions created by the AFM tip. The cross-sectional profile of the worn area is labeled with a dashed line in Figure 5c,d. The micrographs reveal that wear damage is significantly more pronounced on the annealed sample than on the as-deposited film (see Figure 5c,d). Furthermore, the volume of materials removed by sliding the AFM tip with a normal load of 5 μN on the specimens was quantified and displayed in Figure 6a. The results indicate that more material was removed from the annealed Ta surface than on the as-deposited film, with volumes of 2.63 × 10^−3^ μm^3^ and 0.6 × 10^−3^ μm^3^, respectively.

To evaluate the frictional behaviors of Ta specimens, the AFM diamond tip was slid across the film surfaces with an applied normal load in the range of 1 and 10 μN on the sample surface. Figure 6b shows that the friction force generally increases with normal load on both as-deposited and annealed specimens’ surfaces. Notably, the friction force recorded on the annealed Ta film was significantly higher than on the as-deposited specimen. Due to the early mechanical failure and substantial wear damage observed on the surface of the annealed sample, no friction force data with a normal load larger than 5 μN were reported in Figure 6b.

While hard surfaces generally produce less mechanical wear damage during sliding contacts, it is surprising that the hard annealed Ta film prepared in this study is more susceptible to wear damage and exhibits high friction, as illustrated in Figure 5 and Figure 6. One possible explanation is that the oxidation of the Ta film during the annealing process increased the film’s compressive residual stress. The XPS spectrum in Figure 1 shows that the thickness of Ta_2_O_5_ increased significantly after annealing at 400 °C. Cheng et al. [39] suggested that oxygen diffuses into Ta film and incorporates at the interstitial site inside the Ta unit cell when heat-treated at annealing temperatures of 400 °C or above. This oxidation process leads to the volumetric expansion of the Ta film, resulting in increased residual compressive stress. Cabral et al. [52] also observed a similar compressive stress increase due to Ta oxidation from thermal treatment processes at 400 °C. They observed that the annealed Ta film is more vulnerable to mechanical failures from external perturbations, such as mechanical contacts. Oxidation-induced increase in film compressive stress has also been identified in vacuum-annealed Ta films on silicon oxide substrates, as reported by Liu et al. [53]. They recorded the degradation of mechanical reliability, including issues like macroscopic scale film peeling and cracking. Therefore, the degradation of the nanotribological behaviors of the annealed Ta films observed in this study is consistent with results reported in the literature.

In addition to the oxidation-induced increase in Ta film compressive stress, our results suggest an additional mechanism leading to the decline in the Ta nanotribological performance. Figure 5 indicates a lack of visual evidence of nanoscale film cracking and delamination failure. This observation suggested that additional unknown nanoscale or atomic scale mechanisms may be playing a role to degrade the nanotribological performances of the annealed Ta film. Future studies should investigate the dependence of surface layer Ta oxide chemical composition on its nanotribological behavior, such as the Ta:O ratio and the molecular structure of oxide film, which may yield a new understanding of material parameters that affect Ta wear performance at the nanoscale.

## 4. Conclusions

In this study, we investigated the impact of 400 °C anneals on the chemical composition and nanotribological and small-scale mechanical properties of Ta thin films. Nanoindentation results indicate that the elastic modulus and hardness of as-deposited Ta film increased from 175.2 ± 12.3 GPa and 12.0 ± 1.4 GPa to 213.1 ± 12.7 GPa and 13.1 ± 1.3 GPa after annealing. However, our results show that despite the increases in elastic modulus and hardness, the nanotribological wear performances of the Ta were degraded significantly. The wear volume produced by the sliding contact of a diamond top on the annealed Ta film was four times larger than that of the as-deposited counterpart. Additionally, the sliding friction experienced by the diamond tip under 5 μN normal load was more pronounced on the annealed specimen. It is believed that the degradation of the wear performance of the annealed sample may be linked to surface oxidation of Ta film at elevated temperatures, which resulted in increased compressive residual stress and reduced mechanical integrity under shear deformation. Additional nanoscale mechanisms may also lead to the degradation of the nanotribological performance in the annealed Ta films.

## Figures and Tables

**Table 1:** Nanoindentation hardness and elastic modulus of bulk fused silica, bare silicon substrate, as-deposited Ta film, and Ta film annealed at 400 °C. Fused silica and silicon substrate results are averaged values from measurements between indentation depths of ~15 nm and ~300 nm. The Ta film results are obtained at an indentation depth of ~15 nm, ~10% of the Ta film thickness.

	Elastic Modulus, E (GPa)	Hardness, H (GPa)
Bulk fused silica	72.8 ± 1.6	8.7 ± 0.2
Bare Si substrate	155.6 ± 4.2	10.0 ± 1.0
As-deposited Ta film	175.2 ± 12.3	12.0 ± 1.4
Annealed Ta film	213.1 ± 12.7	13.1 ± 1.3

**Table 2:** Surface roughness results of as-deposited Ta film and film annealed at 400 °C.

	Root Mean Square, RMS (nm)	Mean Roughness (nm)
As-deposited	0.7 ± 0.1	0.6 ± 0.1
Annealed	0.5 ± 0.04	0.4 ± 0.04

## Data Availability

The data presented in this study are available upon request from the corresponding author.
